# Supplementary material for: Mechanisms governing the pioneering and redistribution capabilities of the non-classical pioneer PU.1
Source: Nat Commun. 2020 Jan 21;11:402. doi: 10.1038/s41467-019-13960-2 (PMC6972792; doi:10.1038/s41467-019-13960-2)
Supplement: Supplementary file 7 — Source data [file 41467_2019_13960_MOESM7_ESM.zip › Source_Data/FigureS3/FigureS3H_MotifScanOutput/homerResults/motif9.similar.html]

motif9

## Information for motif9

C
G
T
A
C
G
T
A
T
G
A
C
A
G
T
C
T
A
C
G
G
T
C
A
G
T
C
A
C
G
T
A
A
T
C
G
G
A
C
T
  
Reverse Opposite:  

C
T
G
A
T
A
G
C
G
C
A
T
A
C
G
T
C
A
G
T
A
T
G
C
T
C
A
G
A
C
T
G
A
C
G
T
C
G
A
T
  

|  |  |
| --- | --- |
| p-value: | 1e-83 |
| log p-value: | -1.915e+02 |
| Information Content per bp: | 1.645 |
| Number of Target Sequences with motif | 794.0 |
| Percentage of Target Sequences with motif | 20.54% |
| Number of Background Sequences with motif | 4291.1 |
| Percentage of Background Sequences with motif | 9.58% |
| Average Position of motif in Targets | 281.8 +/- 299.0bp |
| Average Position of motif in Background | 210.9 +/- 140.1bp |
| Strand Bias (log2 ratio + to - strand density) | -0.1 |
| Multiplicity (# of sites on avg that occur together) | 1.11 |
| Motif File: | file (matrix) reverse opposite |

### Similar de novo motifs found

|  |  |  |  |  |  |  |  |
| --- | --- | --- | --- | --- | --- | --- | --- |
| Rank | Match Score | Redundant Motif | P-value | log P-value | % of Targets | % of Background | Motif file |
| 1 | 0.796 | T C A G T G C A T G C A C G T A A T G C A G C T C T A G G T C A G T C A C G T A T A C G G A C T | 1e-82 | -189.438929 | 6.83% | 1.39% | motif file (matrix) |
| 2 | 0.854 | C T A G A T G C G A C T A C G T A C G T A T G C C T A G A T C G A G C T G A C T A G C T | 1e-66 | -152.942338 | 13.94% | 5.89% | motif file (matrix) |
